# Ultrasound assessment of insular development in adequate-for-gestational-age fetuses and fetuses with early-onset fetal growth restriction using 3D-ICRV technology

**DOI:** 10.3389/fmed.2024.1393115

**Published:** 2024-10-09

**Authors:** Jinfeng Xue, Jinluan Xue, Yanhui Ru, Ge Zhang, Hong Yin, Dequan Liu

**Affiliations:** ^1^School of Medical Imaging, Shandong Second Medical University, Weifang, China; ^2^Department of Ultrasound, Shandong Provincial Maternal and Child Health Care Hospital, Jinan, China; ^3^Medical Department, Liaocheng Third People’s Hospital, Liaocheng, China

**Keywords:** insula, 3D-ICRV, fetus, fetal growth restriction, cortical development and maturity

## Abstract

**Objective:**

This study aimed to evaluate the growth trajectory of the insula in adequate-for-gestational-age (AGA) and early-onset fetal growth restriction (FGR) fetuses and analyze the difference between the two groups using three-dimensional inversion crytal and realistic vue technique (3D-ICRV).

**Methods:**

Singleton pregnant women, with a gestational age ranging from 20 to 32^+6^ weeks, who underwent routine examinations at Shandong Maternal and Child Care Hospital between March 2023 and December 2023 were included. The participants were divided into two groups: the FGR and AGA fetuses. Three-dimensional volumes were obtained using transabdominal ultrasound in the transverse section of the fetal hypothalamus based on different gestational ages. 3D-ICRV rendering technology was used for 3D imaging of the fetal insula. Volumes with a clear display of the insula were selected. We observed the morphology of the insula, and measured the area and circumference of the insula. By evaluating the growth trajectory of the insula in AGA and FGR fetuses, differences in insular development between the two groups were compared.

**Results:**

Overall, 203 participants were included in this study, with 164 and 39 in the AGA and FGR groups, respectively. The 3D volumes were successfully acquired, and the area and circumference of the insula were measured using 3D-ICRV imaging technology. We found that as gestational age increased, the area and circumference of the insula gradually increased and showed positive correlations with the gestational age, with no significant changes in morphology. The growth rate of insular area and insular circumference in the FGR group is slower than that in the AGA group (insular area: 0.15 vs 0.19 cm^2^ / week, insular circumference: 0.25 vs 0.28 cm / week). The area and circumference of the insula in the FGR group were significantly different from those in the AGA group (insular area: *p* = 0.003, insular circumference: *p* = 0.004).

**Conclusion:**

The measured values of the insula using 3D-ICRV identify the differences in insular development between the FGR and AGA fetuses. The findings of this study have important implications for the prenatal evaluation of cortical development and maturity in FGR fetuses and further clinical consultation and management.

## 1 Introduction

Fetal growth restriction (FGR) is the phenomenon in which fetal growth does not reach its genetic potential due to the influence of maternal, placental, and fetal pathological factors. The FGR is diagnosed annually worldwide, with about 30 million cases (1). Infants born with FGR not only exhibit cognitive, motor, and language delays during infancy and early childhood but also continue to experience impaired cognitive function during school age (2). Studies on the neurodevelopment of early-onset FGR offspring suggest that FGR is responsible for postnatal motor, cognitive, and behavioral abnormalities (3). The insular cortex, a brain region, plays a crucial role in human cognitive function (4). Insula is the first cortical region to differentiate and develop. The cortical region of the insula begins to form in the 6th week of embryonic development (5). Evidence suggests that the maturation and vascularization of the cortical surface of the brain begins in the insular region, indicating that this region is central to cortical development (6). Abnormal, delayed, or insufficient gyrification can lead to cortical anomalies. This may be associated with secondary factors affecting brain development, such as intrauterine growth restriction or congenital heart disease (7). In addition to helping with the early detection of cortical developmental anomalies, research on the insula development of FGR fetuses can help predict the neurodevelopmental outcomes of these cases and help implement the best clinical management strategies, potentially reducing or eliminating abnormal neurodevelopment.

Most of the previous information on the development of the fetal insula cortex comes from descriptive anatomical studies and MRI of gross brain specimens. But MRI examination of fetal brain is time-consuming and expensive, which is not recommended for routine examination when there is no abnormal indication (8). Two-dimensional ultrasound can only observe the insula in fixed axial planes, and cannot display the overall morphological changes of insula. Therefore, it is of great clinical value to explore new methods for ultrasound imaging the insula of fetuses. This study used a novel three-dimensional inversion crytal and realistic vue technique (3D-ICRV) to observe the insula in adequate-for-gestational-age (AGA) and FGR fetuses. Chen et al. (9) stumbled upon the use of multiple 3D imaging techniques to realistically display the sulci and gyrus on the surface of the fetal brain while studying fetal brain 3D imaging, and named this technique 3D-ICRV. By collaborating with Samsung to package and combine the above technologies, this technology has been applied in a one click manner in the W10 color Doppler ultrasound instrument. The visualization method can display the insular cortex, which is hidden in the depths of the cerebral hemisphere, as an anatomical entity. It allows for a more vivid observation of changes in the fetal insula morphology and size during different gestational weeks, providing an opportunity for further evaluation of cortical development *in utero*. Chen et al. (9) use 3D-ICRV imaging technology to observe the sulcus gyrus on the surface of the appropriately grown fetal cerebral hemisphere, and this technique is used to demonstrate the cortical developmental changes of the appropriately grown fetuses associated with gestational age. Yi et al. (10) use 3D-ICRV imaging technology to observe the lateral surface fissure of the fetal brain from 20 to 32+6 weeks, the lateral fissure development pattern was evaluated and established the reference value range of the uncovered area and circumference of the insula. These studies confirm that this technique has the advantages of good consistency and repeatability, simple operation, short time-consuming, and low cost.

In this study, we novelly used a 3D-ICRV imaging technology to spread the insula of the fetal brain like entities. Then they were used to evaluate the developmental pattern of the insula in AGA and early-onset FGR fetuses between 20 and 32^+6^ weeks of gestation, which was further compared with the two groups by measurements of the area and circumference of the insula and observed the difference between the two groups. To our knowledge, this is the first study to compare the insula of early-onset FGR fetuses and AGA fetuses using 3D-ICRV technology.

## 2 Materials and methods

### 2.1 Study participants

This prospective cross-sectional study was conducted on pregnant women who underwent routine prenatal examinations at Shandong Maternal and Child Care Hospital between March 2023 and December 2023. All participants were Asians. This study was approved by the Medical Ethics Committee of Shandong Maternal and Child Care Hospital, and informed consent was obtained from all participants (No. 2024-039).

#### 2.1.1 Inclusion criteria for the AGA group

Singleton pregnant women with a gestational age between 20 and 32+6 weeks and fetuses without structural or chromosomal abnormalities were recruited in the AGA group.

#### 2.1.2 Inclusion criteria for the early-onset FGR group

We recruited singleton pregnant women with a gestational age between 20 and 32^+6^ weeks and early-onset FGR fetuses. Using crown-rump length (CRL) as a measure of gestational age in early pregnancy (11). For early FGR (<32 weeks), (1) abdominal circumference (AC) < 3(rd) centile, estimated fetal weight (EFW) < 3(rd) centile (2) absent end-diastolic flow in the umbilical artery (UA). (3) AC or EFW < 10(th) centile combined with a pulsatility index (PI) > 95(th) centile in either the UA or uterine artery (12).

#### 2.1.3 Exclusion criteria

We excluded ultrasound images with poor quality or presence of fetal anomalies. All pregnant women underwent systematic ultrasound screening during the second trimester of pregnancy to exclude fetal anomalies. During follow-up, abnormal conditions such as birth weight and 1-min Apgar score of FGR fetuses were observed after delivery.

### 2.2 Ultrasound equipment and examination methods

#### 2.2.1 Ultrasound equipment

Samsung HERA W10 ultrasound device (Samsung Medison Co., Ltd., Seoul) and an abdominal convex transducer with a frequency range of 1–8 MHz.

#### 2.2.2 Examination methods

##### 2.2.2.1 Fetal biometric parameters and Doppler waveforms of blood flow

All cases were evaluated using the last menstrual period or CRL as a measure of gestational age in early pregnancy. A systematic fetal examination was performed to exclude fetal anomalies. Standard biometric measurements, including biparietal diameter (BPD), head circumference (HC), AC, and femur length (FL), were obtained for all fetuses, and EFW was calculated. Doppler blood flow parameters were additionally measured for FGR cases, including umbilical artery pulsatility index (UAPI), fetal middle cerebral artery pulsatility index (MCAPI), fetal ductus venous waveform, and bilateral uterine artery PI of the mother, and the cerebroplacental ratio (CPR) was calculated.

##### 2.2.2.2 The 3D-ICRV software was used to obtain visual images of the insula

According to the International Society of Ultrasound in Obstetrics and Gynecology (ISUOG) practice guidelines (13) for sonographic examination of the fetal central nervous system, a detailed sonographic examination of the fetal brain was performed. (1) The initial two-dimensional (2D) plane was set as the transverse section through the fetal thalami, displaying the corpus callosum and the cavum septi pellucidi. Appropriate scanning depth was selected according to gestational age, and the image was magnified to display the insular cortex. (2) The 3D volumetric scanning mode was applied with the fetus being static. The “brain” mode was selected, and the scanning angle was adjusted according to gestational age, typically around 80°. The scanning quality was set to the highest level, and the transducer was placed tightly against the maternal abdominal wall to acquire the volume. (3) After acquiring the volumetric data, the beam arrow was set from bottom to top in the rendering plane (Render Setup). The Crystal Vue imaging mode was selected in the control panel, and Realistic Vue imaging and Inversion modes were overlaid.

**FIGURE 1:**
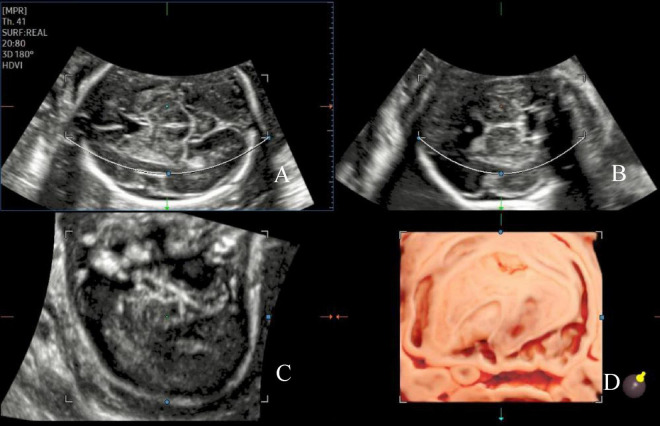
Image of the AGA fetal insula at 24 weeks of gestation. The initial three spatially perpendicular planes for the assessment of the insula (IN), with the basic imaging plane being the transthalamic plane **(A)**. The three orthogonal views **(A–C)** correspond to the coronal, sagittal, and axial slices, respectively, and the single images from each of the three orthogonal views are visualized **(D)**.

**FIGURE 2:**
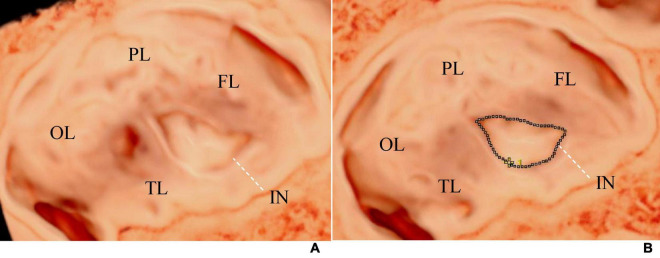
**(A)** Shows the visualization image of the AGA fetal insula at 32 weeks of gestation, and **(B)** demonstrates the method of measuring the insular area and circumference. The tracing method was used to draw a line along the edge of the insula to measure its area and circumference. The dashed line represents the tracing line. FL, frontal lobe; PL, parietal lobe; OL, occipital lobe; TL, temporal lobe; IN, insula.

The initial image comprised three spatially perpendicular planes, namely the A- (transverse plane), B- (coronal plane), and C-planes (sagittal plane). The ROI cropping line below the A-plane was adjusted to be located at the outer edge of the insular cortex, with the same curvature and direction as the insular cortex, to ensure that the outline of the insula and its gyri and sulci were presented accurately on the observed surface. To obtain the visual 3D images of the insula from the far end of the transducer, the light source was switched, and the trackball was moved to adjust the appropriate light source angle, thereby increasing the shading of the cortical folds (Figure 1) (4). Finally, the images were stored for further measurement and post-processing. The marking method of Samsung HERA W10 ultrasound device was used on the 3D image to draw a line along the boundary of the insula, and the insular circumference and area was measured (Figure 2). All ultrasound images were obtained using standardized methods by a skilled sonographer. The same examiner performed all measurements. During measurement, the image was enlarged to 2/3 of the entire screen to display the boundary of the insula. Three measurements were taken, and the average of the three measurements was recorded.

### 2.3 Analysis of insula development in FGR and AGA groups

To measure the changes in fetal insular circumference and area with gestational age in the FGR and AGA groups, linear fitting was performed using Python (version 3.10.12). The fitting curve and insula development rate were output. The mean and standard deviation of the insular area and circumference at different gestational ages were obtained. And the homogeneity of variance between AGA and FGR samples for each gestational age group was tested using Levene’s test. Then *t*-test was used for test differences between AGA and FGR samples for each gestational age group. *P*-value < 0.05 was considered statistically significant.

### 2.4 Statistical analysis

Statistical analysis was performed using SPSS 22.0 software (SPSS Inc, Chicago, IL, USA). To eliminate the impact of gestational age, the insular circumference and area measurements were transformed into standard normal distributions (Z-scores) using the formula Z = (observed value - mean) / standard deviation, which were labeled as Z-scores for insular circumference and area, respectively. Independent samples *t*-test was performed on the Z-scores of insular measurements between the AGA and FGR groups, as well as between the left and right insula in the FGR group. As the insular circumference-to-HC ratio and insular area-to-HC ratio followed a normal distribution, the *t*-test was performed. A *P*-value < 0.05 was considered statistically significant.

## 3 Results

### 3.1 General information and Doppler flow examination results

Five cases were excluded due to poor image quality and fetal structural malformations. Overall, 203 participants were included, with 164 and 39 in AGA and FGR groups, respectively (Supplementary Table 1). All enrolled fetuses were grouped into 13 groups according to gestational age, from 20 to 32+6 weeks. Each pregnant woman was scanned once, and 203 cases successfully obtained the fetal brain volume that could clearly display the rendered images of the insula. Three optimal volumes per fetus were selected for analysis. Table 1 presents the clinical characteristics of the pregnant women in the FGR and AGA groups and Comparison of Doppler blood flow parameters between FGR and AGA group. No significant differences in maternal age and height were observed between the two groups (*P* = 0.288, *P* = 0.131), whereas significant differences in weight and body mass index were observed (*P* = 0.028, *P* = 0.012). Moreover, gestational age of FGR group and AGA group is 27.38 ± 3.35 weeks, and 26.00 ± 3.49 weeks, respectively (Table 1). In the FGR group, 14 patients had abnormal umbilical artery (UA) spectrum morphology, including three disappeared diastolic blood flow, one reversed diastolic blood flow, and 10 increased PI. The CPR and MCAPI were significantly lower.

**TABLE 1:** Demographic characteristics and middle cerebral artery (MCA) blood flow indexes of the study groups.

	FGR (*n* = 39)	AGA (*n* = 164)	*P*
Age of the pregnant woman (years)	30.10 ± 4.93	30.39 ± 4.13	0.288
Height (cm)	162.33 ± 5.21	163.15 ± 4.56	0.131
Body weight (kg)	71.10 ± 14.07	65.73 ± 9.53	0.028
BMI	27.18 ± 5.65	24.70 ± 3.51	0.012
Gestational age at the time of measurement	27.38 ± 3.35	26.00 ± 3.49	0.025
MCAPI	1.78 ± 0.30	2.01 ± 0.23	<0.0001
CPR	1.65 ± 0.35	1.85 ± 0.20	<0.0001

Data are shown as mean ± SD. FGR, fetal growth restriction; AGA, adequate-for-gestational-age; BMI, body mass index; MCA, middle cerebral artery; PI, pulse index; PR, cerebral–placental ratio.

### 3.2 Analysis of the developmental characteristics, area, and circumference measurements of the insula in the fetal brain of the AGA and FGR group

**FIGURE 3:**
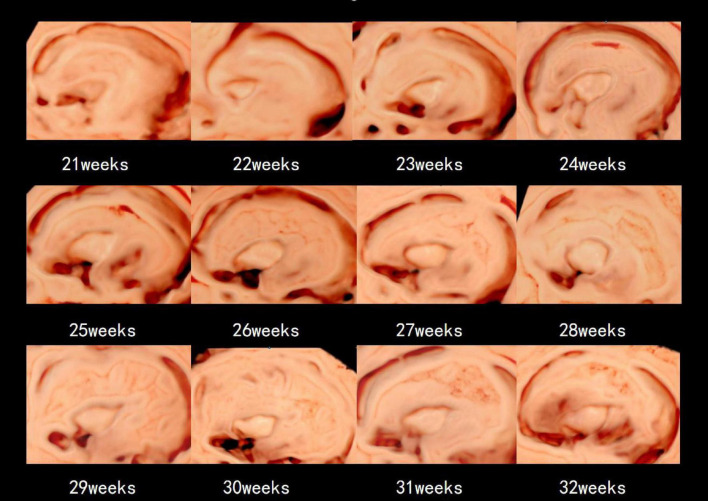
The 3D-ICRV sonographic characteristics of the insular cortex. Images are taken from individual fetuses rather than longitudinal assessment in one fetus.

**FIGURE 4:**
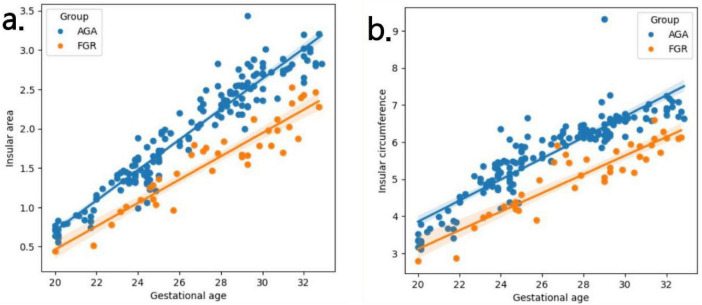
**(a,b)** Fitting curves of fetal insula changes with gestational age. The weekly increase in insular area of a fetus in AGA group and FGR group is 0.19 vs 0.15 (cm2), respectively. The weekly increase in insular circumference of a fetus is 0.28 vs 0.25 (cm), respectively.

Table 2 presents the mean, standard deviation and *P*-value of the insular area and circumference measurements at different gestational ages in the AGA and FGR fetuses. The detailed 3D-ICRV sonographic characteristics of the insular cortex in the fetal brain at different gestational ages are shown in Figure 3. The insular cortex is displayed as an inverted triangle using the 3D-ICRV technology. With increasing gestational ages, the shape of the insular cortex did not show significant changes, whereas the area and circumference gradually increased, which were positively correlated with gestational age. As shown in Figures 4a, b, the weekly increase in insular area of a fetus in AGA group and FGR group is 0.19 vs 0.15 (cm2), respectively, and the weekly increase in insular circumference of a fetus is 0.28 vs 0.25 (cm), respectively.

**TABLE 2:** Insular circumference and area measurements at different gestational ages in the AGA group and FGR group.

	Insular area			Insular circumference		
GA	FGR	AGA	*P*	FGR	AGA	*P*
20-20^+6^	0.44 ± 0.04	0.70 ± 0.10	0.0002	2.79 ± 0.07	3.39 ± 0.27	0.0008
21-21^+6^	0.52 ± 0.02	0.88 ± 0.07	<0.0001	2.87 ± 0.11	3.80 ± 0.23	<0.0001
22-22^+6^	0.78 ± 0.01	1.19 ± 0.13	<0.0001	3.68 ± 0.07	4.61 ± 0.32	0.0003
23-23^+6^	0.98 ± 0.05	1.42 ± 0.14	<0.0001	4.0 ± 0.12	5.04 ± 0.31	<0.0001
24-24^+6^	1.15 ± 0.12	1.45 ± 0.26	0.0003	4.26 ± 0.14	5.15 ± 0.56	<0.0001
25-25^+6^	1.25 ± 0.22	1.76 ± 0.18	<0.0001	4.49 ± 0.49	5.76 ± 0.37	<0.0001
26-26^+6^	1.73 ± 0.13	1.93 ± 0.19	0.026	5.68 ± 0.28	5.84 ± 0.36	0.36
27-27^+6^	1.65 ± 0.15	2.33 ± 0.29	<0.0001	5.26 ± 0.39	6.2 ± 0.3	<0.0001
28-28^+6^	1.84 ± 0.15	2.37 ± 0.22	0.0002	5.54 ± 0.17	6.25 ± 0.31	0.0004
29-29^+6^	1.73 ± 0.24	2.55 ± 0.34	<0.0001	5.25 ± 0.39	6.61 ± 1.14	<0.0001
30-30^+6^	1.98 ± 0.15	2.67 ± 0.25	<0.0001	5.72 ± 0.36	6.54 ± 0.25	<0.0001
31-31^+6^	2.17 ± 0.32	2.78 ± 0.16	<0.0001	6.05 ± 0.39	6.79 ± 0.24	<0.0001
32-32^+6^	2.40 ± 0.18	2.91 ± 0.22	<0.0001	6.11 ± 0.16	6.91 ± 0.22	<0.0001

Data are shown as mean ± SD or percentage as appropriate. FGR, fetal growth restriction; AGA, adequate-for-gestational-age; GA, gestational age.

### 3.3 Comparison of the results of insular cortex measurements in the FGR group with those of the AGA group and the observation of the perinatal outcome of the study groups

Our results showed significant differences in insular circumference and area measurements between the FGR and AGA groups, with *P*-values of 0.004 and 0.003, respectively. Although the average gestational age in the FGR group was greater than that in the AGA group (27.38 vs 26.00), its insular area and circumference were still significantly smaller than those in the AGA group. Even after adjusting for HC, the differences between the two groups remained statistically significant (*P* < 0.001) (Table 3). The comparison of insular area and circumference between the left and right hemispheres in the FGR group (20 cases for the left hemisphere; 19 cases for the right hemisphere) showed no statistically significant differences (area: *P* = 0.893; circumference: *P* = 0.989).

**TABLE 3:** Insular features of the FGR group and the AGA group for 20–32+6 weeks.

	FGR (*n* = 39)	AGA (*n* = 164)	*t*	*P*
Insular circumference(Z-score)	−0.419 ± 0.883	0.996 ± 1.000	2.965	0.003
Insular area(Z-score)	−0.355 ± 0.781	0.084 ± 1.029	2.957	0.004
Insular circumference/head circumference	2.123 ± 0.162	2.302 ± 0.183	5.588	<0.0001
Insular area/head circumference	0.662 ± 0.144	0.766 ± 0.190	3.772	<0.0001

We followed the perinatal outcomes of all FGR fetal cases and 40 AGA fetuses. FGR fetuses were delivered earlier with higher rates of emergency cesarean section, labor induction, and lower Apgar scores at 5 min of life (Table 4).

**TABLE 4:** Perinatal outcome of the study groups.

	FGR (*n* = 39)	AGA (*n* = 40)	*P*
GA at birth (wks)	35.4 ± 3.4	39 ± 1.0	<0.0001
Birth weight (g)	1828 ± 732	3162 ± 359	<0.0001
Emergency cesarean section	31%	7.5%	<0.0001
Apgar score less than 7 at 5 min	15%	2.5%	<0.0001

Data are shown as mean ± SD or percentage as appropriate. FGR, fetal growth restriction; AGA, adequate-for-gestational-age; GA, gestational age.

## 4 Discussion

In this study, we utilized sonography with 3D-ICRV technology a total of 203 pregnant women (AGA: 164, FGR: 39) enrolled at 20-32^+6^ weeks were scanned, and provided a visual and 3D display of the full view of the fetal brain’s insular cortex. We divided 203 pregnant women into 13 groups by weekly gestational age and measured the circumference and area of the insula separately. We evaluated the growth trajectory of the insula in AGA and FGR fetuses, and identified differences in insular development between the two AGA and FGR groups (11). By observing the insular cortex at different gestational ages, we found that the insular cortex appeared as an inverted triangle, and its circumference and area showed significant positive correlations with gestational age, increasing with gestational age but without significant changes in shape. In addition, we also found a difference in the growth rate of the insula between FGR and AGA with insular area increasing of 0.15 vs 0.19 cm^2^ per week and insular circumference increasing of 0.25 vs 0.28 cm per week. The measured values of the insula using 3D-ICRV identify the differences in insular development between showed significant differences between the FGR and AGA fetuses. The findings of this study have important implications for the prenatal evaluation of cortical development and maturity in early-onset FGR fetuses and further clinical consultation and management.

This study validates the effectiveness of 3D-ICRV for early-onset FGR fetal monitoring. Early-onset FGR induced cardiovascular redistribution due to placental dysfunction and progressive fetal hypoxia, with uneven fetal growth and cerebral protective effects (3). Several studies have shown that prenatal ultrasound finding FGR fetuses with brain protective effects have poorer neurodevelopment than FGR fetuses with no brain protective effects (14). According to the literature, the hippocampus is particularly vulnerable in cases of intrauterine hypoxia (15, 16), and the phylogenetic origin of the hippocampus is similar to that of the insular cortex (17). Therefore, it can be inferred that the insular cortex may also be susceptible to relatively sustained malnutrition and hypoxia, resulting in some changes (18). Therefore, obtaining reliable data is able to assess the severity of fetal hypoxia, which in turn allows selective application to appropriate intrauterine management (19). And postnatal treatment options, the development of such intrauterine imaging markers provides a new approach for the clinical monitoring of FGR. For instance, in FGR fetuses exhibiting abnormal insula development, it becomes crucial to accord heightened attention to the maturation of their nervous system and institute corresponding neuroprotective strategies. These measures may encompass, but are not limited to, augmenting nutritional support, enhancing placental functionality to bolster blood and oxygen perfusion, and administering specific pharmacotherapies or interventions aimed at nurturing or safeguarding neuronal development.

This work further shows the insular development procession of early-onset FGR fetuses and provides a reference value for monitoring FGR using 3D-ICRV. Previous studies on the insular cortex have mainly focused on late-onset SGA fetuses with a fetal weight below the 10th percentile (18–21). However, it should be noted that SGA fetuses include both pathological FGR fetuses and some healthy fetuses (18). Studies have shown that the lower the cutoff values for AC and EFW, the higher the risk of true FGR (22). In this study, fetuses at 20-32^+6^ gestational weeks with severe early-onset FGR defined by EFW and/or AC less than the 3rd percentile, with or without Doppler abnormalities or EFW and/or AC less than the 10th percentile combined with Doppler abnormalities (12), were included to reduce the confounding effects from healthy fetuses. After adjusting for HC, we could differentiate between the decreases in biometric measurements of the insular cortex due to FGR itself and those due to a smaller head, confirming that severe FGR fetuses showed differences in early biometric measurements of the insular cortex.

This work clarifies the differences in insular development between early-onset FGR fetuses and AGA fetuses. Numerous studies exist on the insular cortex in fetuses born with FGR and small for gestational age (SGA). In 2014, a study by Egaña-Ugrinovic et al. (18) using MRI found a significant reduction in cortical thickness and volume of the insula in fetuses with late-onset SGA compared to the AGA group, which was associated with poorer neurobehavioral outcomes. Putra et al. (20) used 2D ultrasound to describe the area and circumference of the hypoechogenic insular zone (HIZ) on the cranial axis plane in FGR and SGA fetuses and found no significant difference between the two groups. However, the circumference of the HIZ was increased compared to the appropriate for gestational age (AGA) fetuses. The results of our study confirmed statistically significant differences in both the area and circumference of the insular cortex between the FGR fetuses and AGA fetuses. The insular area and circumference were both smaller in FGR fetuses than those in AGA fetuses, and these differences remained statistically significant even after adjusting for HC. The reasons for the different research results are as follows: (1) different examination techniques (MRI vs. 2D ultrasound vs. 3D-ICR ultrasound); (2) differences in the morphology and methods of insular cortex measurement; and (3) differences in the onset time and severity of intrauterine growth restriction. It has been demonstrated that severe hypoxia can inhibit synaptic plasticity, leading to delayed neuronal development, neuronal death, and neural damage. However, mild hypoxia can enhance neuronal proliferation and maturation (23, 24).

This work firstly used 3D-ICRV technology to monitor the overall morphology and developmental patterns of the insular cortex in AGA and early-onset FGR fetuses. 3D-ICRV imaging technology is a novel rendering technique for 3D volume measurement developed by Samsung Medison Co., Ltd. (Seoul, Korea). Previous studies have reported the use of 3D-ICRV technology for the prenatal diagnosis of midgut volvulus (25), diagnosis of the abnormally invasive placenta (26), and sonographic visualization of the fetal esophagus (27). These studies have demonstrated the advantages of 3D-ICRV technology for diagnosis. Currently, there is no literature describing the overall morphological, developmental patterns of the insular cortex in the AGA and FGR fetuses using 3D-ICRV technology. Previous studies on the fetal cerebral cortex have mostly used 2D ultrasound, conventional 3D ultrasound, and MRI examinations. MRI has been considered the gold standard for non-invasive fetal brain structure research. However, it is unsuitable for continuous and repetitive monitoring of FGR fetuses in clinical practice owing to its long scanning time and high cost. The 2D and conventional 3D ultrasounds have limited observational views and provide limited information about the overall morphology of the insular cortex. Meanwhile, 3D-ICRV imaging technology can provide a complete view of the insular cortex and display the fine contours between the insular cortex and the surrounding brain structures realistically. The entire procedure takes approximately 5 min, and it is simple and time-efficient with high feasibility and reproducibility.

Our research also has limitations. Firstly, the samples collected during the non-primary prenatal screening period was relatively insufficient, resulting in an uneven distribution of gestational ages in the sample. In fact, the occurrence of FGR at small gestational age can lead to a decrease in live birth rate. We excluded infants who did not live, resulting in a different distribution of gestational age between the FGR group and the AGA group. Secondly, owing to the influence of near-field artifacts caused by the proximal skull, only the far side of the brain hemisphere could be observed using the transducer. Thirdly, the data for this study were obtained by a ultrasound doctor using triple replicate measurements, and the reproducibility among different ultrasound doctor ought to be rigorously evaluated in subsequent larger studies. Lastly, long-term follow-up observations are needed to investigate the relationship between the differences in insular biometric measurements in early-onset FGR fetuses and their postnatal neurodevelopmental outcomes.

Summarily, the 3D-ICRV is a novel method for observing the development of the fetal insular cortex. To provide a theoretical basis for the clinical management and treatment of FGR. It is simple to operate, time-efficient, and can be a valuable tool in clinical practice.

## Data Availability

The datasets presented in this study can be found in online repositories. The names of the repository/repositories and accession number(s) can be found in this article/Supplementary material.
