# Supplementary material for: Ultrasound assessment of insular development in adequate-for-gestational-age fetuses and fetuses with early-onset fetal growth restriction using 3D-ICRV technology
Source: Front Med (Lausanne). 2024 Oct 9;11:1393115. doi: 10.3389/fmed.2024.1393115 (PMC11496279; doi:10.3389/fmed.2024.1393115)
Supplement: Supplementary file 1 [file Data_Sheet_1.docx]

**Supplementary Material**

Table S1. Sample size at different gestational ages in the AGA group and the FGR group.

| GA | FGR | AGA |
| --- | --- | --- |
| 20-20^+6^ | 1 | 12 |
| 21-21^+6^ | 1 | 8 |
| 22-22^+6^ | 1 | 5 |
| 23-23^+6^ | 2 | 20 |
| 24-24^+6^ | 5 | 21 |
| 25-25^+6^ | 3 | 13 |
| 26-26^+6^ | 2 | 9 |
| 27-27^+6^ | 4 | 10 |
| 28-28^+6^ | 1 | 18 |
| 29-29^+6^ | 6 | 21 |
| 30-30^+6^ | 4 | 9 |
| 31-31^+6^ | 6 | 67 |
| 32-32^+6^ | 3 | 12 |

Abbreviations: FGR, fetal growth restriction; AGA, adequate-for-gestational-age; GA, gestational age.


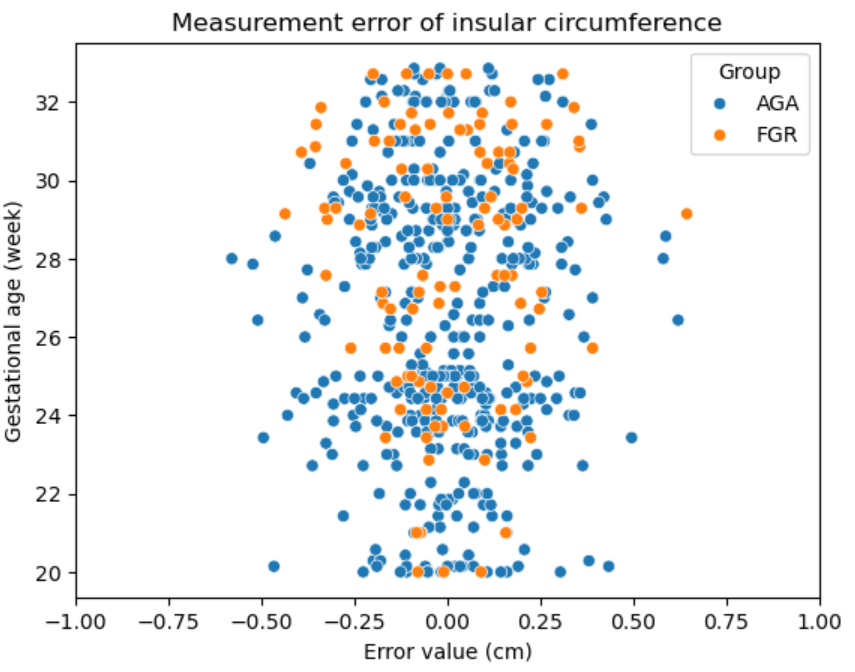

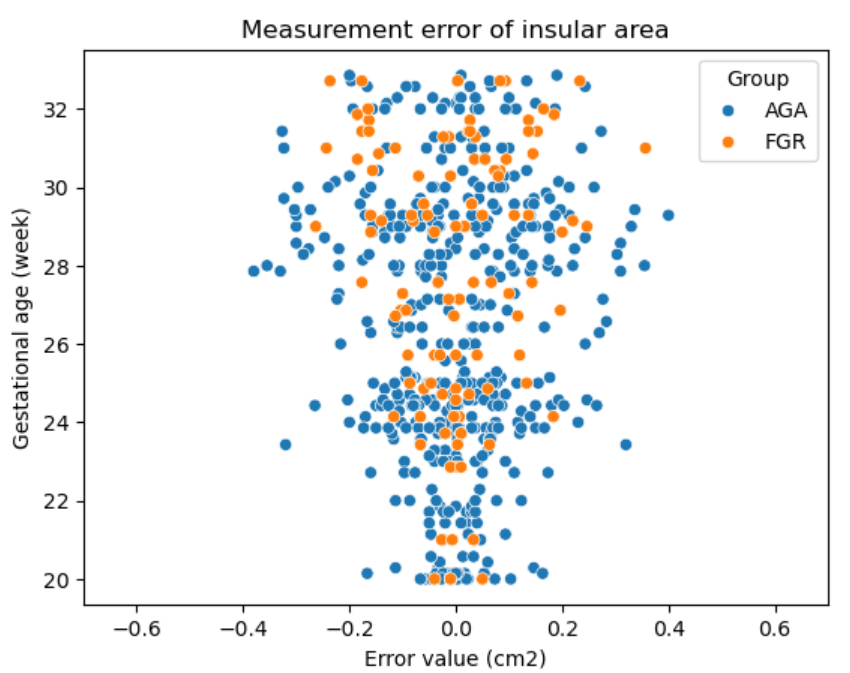


Figure S1. The measurement error values of the AGA and FGR groups by the same ultrasound doctor using the triple repeat measurement method. The y-axis represents gestational age.The measurement error for the circumference of the insula is generally observed to be within 0.75 centimeters, while the measurement error for the area of the insula typically remains within 0.4 square centimeters. It is noteworthy that the measurement error does not exhibit any variation based on grouping or gestational age.
